# Serum Inflammatory Markers and Integrity of the Superior Longitudinal Fasciculus and the Inferior Longitudinal Fasciculus in Schizophrenia, from Prodromal Stages to Chronic Psychosis—A Cross-Sectional Study

**DOI:** 10.3390/jcm12020683

**Published:** 2023-01-15

**Authors:** Anna Michalczyk, Ernest Tyburski, Piotr Podwalski, Katarzyna Waszczuk, Krzysztof Rudkowski, Jolanta Kucharska-Mazur, Monika Mak, Katarzyna Rek-Owodziń, Piotr Plichta, Maksymilian Bielecki, Wojciech Andrusewicz, Elżbieta Cecerska-Heryć, Agnieszka Samochowiec, Błażej Misiak, Leszek Sagan, Jerzy Samochowiec

**Affiliations:** 1 Department of Psychiatry, Pomeranian Medical University, 71-460 Szczecin, Poland; 2 Department of Health Psychology, Pomeranian Medical University, 71-460 Szczecin, Poland; 3 Department of Neurosurgery, Pomeranian Medical University, 71-252 Szczecin, Poland; 4 Department of Laboratory Medicine, Pomeranian Medical University, 70-111 Szczecin, Poland; 5 Department of Clinical Psychology, University of Szczecin, 71-017 Szczecin, Poland; 6 Department of Psychiatry, Division of Consultation Psychiatry and Neuroscience, Wroclaw Medical University, 50-368 Wroclaw, Poland

**Keywords:** schizophrenia, first episode psychosis, UHR, superior longitudinal fasciculus, inferior longitudinal fasciculus, diffusion tensor imaging, cytokines, inflammatory markers, inflammation

## Abstract

Chronic subclinical inflammation is believed to be an important factor in the pathogenesis of schizophrenia. Meta-analyses confirm the presence of increased levels of peripheral inflammatory markers (IM) in schizophrenia and its prodromal stages. Peripheral cytokines may affect the brain microstructure through chronic activation of microglia. Disruptions in the integrity of the superior longitudinal fasciculus (SLF) and inferior longitudinal fasciculus (ILF) are commonly seen in patients with schizophrenia spectrum disorders. We therefore attempted to verify in a cross-sectional study whether there is a correlation between levels of peripheral IM and the integrity of these brain regions in healthy controls, from prodromal states and first episode psychosis to long-term schizophrenia. The integrity of white matter was measured using diffusion tensor imaging. Despite a broad analysis of six IM (CRP, IL-6, IL-8, IL-10, TNF-α, and IFN-γ), we did not find any correlations with the integrity of the SLF or ILF in any of the analyzed groups (after correction for multiple comparisons). In conclusion, our study does not support the existence of a link between disrupted levels of peripheral IM and reduced integrity of ILF and SLF in schizophrenia spectrum disorders. However, prospective studies are needed to verify this over a long period of time.

## 1. Introduction

Schizophrenia is a chronic mental disorder with a varied and unpredictable course and response to treatment. The prodromal symptoms of the disorder often appear several years before the onset of full-blown psychosis, and this condition is called the “ultra-high risk state” (UHR) [1]. Early identification and implementation of preventive/treatment strategies can potentially alleviate the course of the disease; however, there is currently a lack of reliable tools to identify an individual’s risk of developing schizophrenia. The duration of untreated psychosis is the most critical prognostic factor in schizophrenia [2]. A full understanding of the biological basis of the development of psychosis would allow us to identify specific changes at the biological level that precede the development of the disease at the clinical level. However, despite many years of research, our knowledge of the biological basis of psychotic disorders remains limited.

Among the various hypotheses regarding the biological basis of such disorders, many studies indicate an association between the development of schizophrenia and chronic subclinical inflammation [3,4,5]. First of all, meta-analyses consistently indicate higher levels of peripheral inflammatory markers relative to control groups in patients with chronic schizophrenia (CS) [6,7,8], first episode of psychosis (FEP) [9,10,11], and in UHR patients [11,12,13]. It should be emphasized, however, that the profile of disrupted cytokines varies between studies, and these differences are not so greatr that it is possible to use them to determine the presence of the disorder in a given individual. Our previous studies have also found associations between peripheral inflammatory markers, especially interleukin 6 (IL-6) and interferon gamma (IFN-γ), and psychotic disorders [14,15]. Other evidence implicating inflammation in the etiology of schizophrenia includes, inter alia: (1) results of postmortem studies indicating activated microglia and elevated expression of inflammatory markers in the brains of schizophrenia patients; (2) genetic associations between schizophrenia and some polymorphisms in regions of DNA coding for immune-related genes; (3) increased risk of schizophrenia in patients with autoinflammatory diseases; (4) the observed influence of non-steroidal anti-inflammatory drugs on reducing the severity of psychotic symptoms; (5) associations between cytokine levels and disease severity and treatment response; and (6) the influence of antipsychotic treatments on levels of certain cytokines [7,10,16,17,18,19,20].

The mechanism behind the link between peripheral inflammation and schizophrenia is unclear. It has been hypothesized, however, that peripheral cytokines may indirectly affect the brain microstructure through activation of microglia [21]. Microglia are immune cells present in the central nervous system and are usually in a quiescent state. Their main function is protection against infections and tissue damage, but they are also engaged in ensuring proper brain development and functioning and may regulate cell apoptosis and affect neural circuits. Peripheral cytokines may access the brain by several pathways and chronically activate microglia, which may disrupt their physiological function and lead to excessive oligodendrocyte precursor apoptosis, which, finally, may affect the volume and integrity of white matter [7,22,23,24,25].

Disruptions of white matter (WM) microstructure are commonly seen in schizophrenia. Diffusion tensor imaging (DTI) allows the visualization of fiber tracts in vivo and thereby the analysis of the integrity of WM in various brain regions. Meta analyses and/or systematic reviews of studies involving this technique have shown reduced integrity of WM, manifesting as reduced fractional anisotropy (FA) and/or increased mean diffusivity (MD), not only in chronic schizophrenia [26], but also in FEP [27,28] and UHR patients [27,29]. Although publications differ in the regions that appear to be affected, the following fiber tracts are frequently indicated: the corpus callosum, cingulum bundle, superior longitudinal fasciculus (SLF), and inferior longitudinal fasciculus (ILF). These regions were also associated with psychotic disorders and/or pathophysiological symptoms in our studies [30,31,32,33].

Our current study focuses on the SLF and ILF. The SLF is a fiber tract that connects the frontal, occipital, parietal, and temporal lobes of the brain and supports visuospatial functions, complex motor planning and behavior, social cognition, and language-related processing and activities [34,35]. The ILF connects the occipital cortex to anterior temporal structures and supports cognitive and affective processes based on visual processing. Reduced integrity of both SLF and ILF has been observed in CS, FEP, and UHR patients [36,37,38]. In our previous study, we found lower FA and higher MD in the right SLF in CS compared to healthy controls (HC). We have also found some correlations between reduced integrity of the right SLF and right ILF and disorganization symptoms in CS. In UHR, we found a negative correlation between MD of the left ILF and negative and general symptoms [33].

There is a limited number of studies analyzing correlations between peripheral inflammation and the integrity of various brain regions, but their results indicate the existence of such associations [19,39,40,41,42,43,44]. However, results differ in terms of the cytokines analyzed and the regions affected. Animal studies have shown that prenatal exposure to inflammation induces changes in the integrity of fronto-striatal-limbic circuits [45]. Our previous study analyzing the corpus callosum region found correlations between reduced integrity and increased levels of IL-6 in healthy controls and IFN-γ in chronic schizophrenia [14]. We have also found some correlations between increased IL-6 and reduced integrity of the cingulum bundle, but they did not remain significant after correction for multiple comparisons [15]. Focusing on the SLF and ILF, associations between reduced integrity of these brain regions and increased peripheral inflammatory markers (IM) have been found in the general population [42] and in patients with bipolar disorder [39]. In schizophrenia, Prasad et al. [19] found such associations for the ILF, while Fu et al. [41] did not find correlations for either the SLF or the ILF.

Given the possible link between disrupted levels of peripheral cytokines and reduced integrity of the SLF and ILF, as well as the limited number of studies and their contradictory results, especially in schizophrenia, this study attempted to verify these associations. We hypothesized that chronic peripheral inflammation is associated with reduced integrity of the SLF and ILF in schizophrenia patients and that these changes might be present in the early stages of the disorder. Thus, the aim of this study was to analyze associations between levels of serum inflammatory markers and the integrity of the SLF and ILF in healthy controls and UHR, FEP, and chronic schizophrenia patients.

## 2. Materials and Methods

### 2.1. Participants and Procedure

A total of 112 individuals participated in the study, including 29 healthy controls with no psychotic disorders (HC) aged 22–48 years old, 12 ultra-high risk (UHR) patients aged 18–32, 19 patients with their first episode of psychosis (FEP) aged 19–37 years, and 52 patients with chronic schizophrenia (CS) aged 25–52. HC were recruited through information spread by students and workers from local universities; the patient groups were recruited from inpatients, day treatment patients, and outpatients at the Clinic of Psychiatry at the Pomeranian Medical University in Szczecin, Poland.

Group-specific inclusion criteria included: (1) for HC, a lack of mental or neurological disorders, verified by psychiatric evaluation and a structured self-report questionnaire; (2) for UHR, meeting the criteria for ultra-high risk of psychosis set out in the Polish translation of the Structured Interview for Prodromal Syndromes (SIPS) [46,47]; (3) for FEP, the presence of previously untreated psychotic symptoms with recent onset and no remission since the beginning of symptoms, as well as being diagnosed with a schizophrenia spectrum disorder (F20–F29) based on the International Classification of Diseases and Related Health Problems (ICD-10) [48,49]; and (4) for CS, a stable mental state (assessed as no acute state of psychosis outbreak in psychiatric evaluation) and having been diagnosed with schizophrenia for at least ten years, with the diagnosis confirmed prior to the start of the study by a properly licensed psychiatrist based on a structured clinical interview in line with the ICD-10 and the Mini-International Neuropsychiatric Interview (MINI) [50]. A stable physical state, the ability to comprehend the test procedures, and the willingness to undergo a neuroimaging procedure were common inclusion criteria. Exclusion criteria were: concomitant psychiatric or neurological disorders based on the ICD-10 (with the exception of the disorder that was the basis for inclusion in a given group), severe somatic conditions (i.e., cancer), a history of cerebral or cranial injury, an evident coincidence of symptoms with use of psychoactive substances, and current inflammatory diseases (i.e., clinical symptoms of current infections; taking anti-inflammatory medicines and autoimmune diseases were not taken into account in the study).

Recruitment of patients took place from August 2017 to June 2021. This was a cross-sectional study without follow-up. Participants underwent a psychiatric examination performed by licensed and properly trained psychiatrists, followed by blood sampling and the DTI procedure. Blood sampling and the DTI procedure were done in the same week—mostly on the same day. In UHR and FEP, the study procedures were performed with up to a four-week delay after admission, due to a lack of compliance in the acute phase of the disease. The patients were undergoing treatment adequate for their health condition. The chlorpromazine equivalent was calculated on the basis of defined daily doses. Functioning was assessed using the Global Assessment of Functioning (GAF) questionnaire [51]. The severity of psychopathological symptoms was measured with the SIPS in UHR and with the Positive and Negative Syndrome Scale (PANSS) [52], taking into account five dimensions of symptoms [32,53] in FEP and CS. The studies were approved by the Bioethical Commission of the Pomeranian Medical University in Szczecin (study KB-0012/49/17/A-1 and KB-0012/159/17/A-1). All participants gave informed written consent to participate in the study.

### 2.2. Inflammatory Markers

The levels of inflammatory markers were measured in serum samples obtained from blood collected in the fasting state between 7 and 9 in the morning. The blood samples were centrifuged within an hour of donation, and the serum was transferred to new tubes and frozen and stored at −80 °C. Samples thawed for the first time were used for all tests.

Analysis was performed using ELISA kits. The assays were performed in batches, containing samples from different study groups, after collecting the appropriate number of samples per plate. The time of sample storage was similar in all groups. To ensure appropriate sensitivity, only high sensitivity assays or kits with a chemiluminescent detection system were used. Assays were performed according to the manufacturer’s protocols, and the final absorbance/luminescence in the samples was measured using a SYNERGY HTX multi-mode reader (BioTek, VT, USA). Best-fit standard curves were generated using a Gen5 Microplate Reader and Imaging Software (BioTek).

According to the protocols, the sensitivities of the kits were: 0.35 pg/mL for IL-6 (Human IL-6 QuantiGlo ELISA Kit; R&D, MN, USA), 0.97 pg/mL for interleukin 8 (IL-8; Human IL-8 QuantiGlo ELISA Kit; R&D), 0.481 pg/mL for TNF-α (Human TNF-α QuantiGlo ELISA Kit; R&D), 0.05 pg/mL for interleukin 10 (IL-10; IL-10 ELISA Kit, HS; Invitrogen, MA, USA), 0.69 pg/mL for interferon gamma (Human IFN-γ HS ELISA Kit; Abcam, Cambridge, UK), and 0.1 mg/L for C-reactive protein (CRP; CRP HS ELISA; DRG).

### 2.3. Acquisition of DTI Data and Image Processing

DTI was performed using a 3.0 Tesla scanner (General Electric Signa HDxt). DTI data were obtained using a single-shot pulse sequence with diffusion-weighted echo planar acquisition and the following imaging parameters: repetition time—11.675 s; echo time—82.80 ms; number of excitations—2; acquisition time—10 min, 19 s; matrix—96 × 96; field of view—240 × 240 mm; slice thickness—3 mm; slice gap—0.50; b value—1000 s/mm^2^; and 25 diffusion gradient directions.

Analysis of DTI was performed with the use of the ExploreDTI tool [54]. The main analysis was preceded by the conversion of DICOM files to the *.nii format. The next step was the verification of the compliance of the sides of the converted images to the originals and correction for signal drift, eddy current, and effects due to motion, followed by the removal of artifacts, like Gibbs ringing. After visual inspection of the data quality, they were used for whole-brain tractography using a constrained spherical deconvolution tracking algorithm.

**Figure 1:**
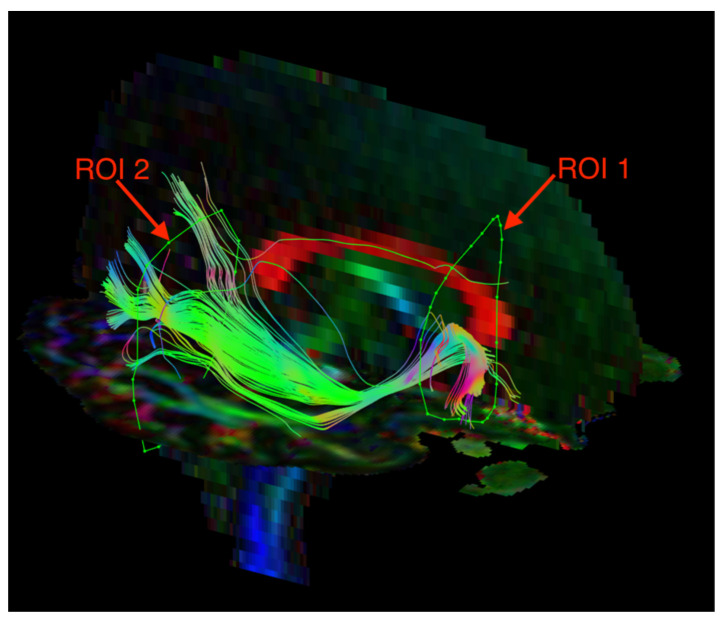
Diffusion tensor tractography of the superior longitudinal fasciculus (SLF) with fractional anisotropy color maps (mid-sagittal plane). Green, red, and blue colors represent fibers running along the axis (anterior–posterior, left–right, and superior–inferior, respectively). Reproduced from [33].

**Figure 2:**
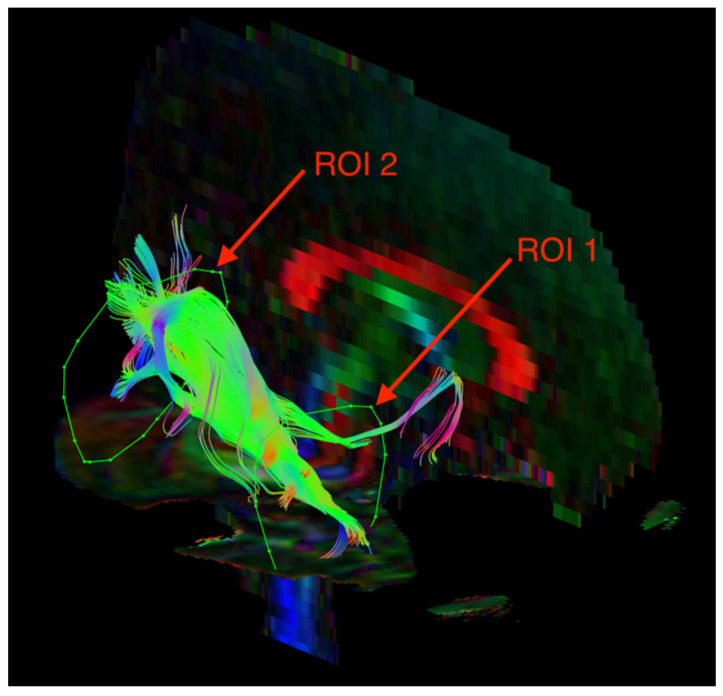
Diffusion tensor tractography of the inferior longitudinal fasciculus (ILF) with fractional anisotropy color maps (mid-sagittal plane). Green, red, and blue colors represent fibers running along the axis (anterior–posterior, left–right, and superior–inferior, respectively). Reproduced from [33].

The visualization of the SLF and ILF was based on an FA color map in the coronal plane. Two regions of interest (ROIs) were used in the analyses of each tract. For the SLF, ROI 1 was at the pole of the frontal lobe in front of the genu of the corpus callosum, and ROI 2 was at the temporal stem (Figure 1). For the ILF, ROI 1 was in the temporal pole, and ROI 2 was at the junction of the temporal and occipital lobes (Figure 2). The parts of the tracts that are not anatomically involved in the SLF/ILF were excluded (as ROInot). The ExploreDTI Descriptive Statistic function was used to automatically calculate the fractional anisotropy and mean diffusivity of the fiber tracts.

### 2.4. Statistical Analysis

Statistical analysis was performed using Statistica 13.3 software (TIBCO Inc). The Shapiro-Wilk test showed that most of the analyzed variables, including levels of inflammatory markers, were non-normally distributed, even after log-transformation; therefore, non-parametric tests were used in all analyses. Group comparisons were performed using a chi-square test for qualitative variables and a Kruskal-Wallis H test for quantitative/ordinal variables. Correlations were analyzed using Spearman’s rank correlation coefficient (ρ). Associations were considered as statistically significant at *p* < 0.05. The Holm-Bonferroni correction was used to analyze the significance of differences/correlations in multiple comparisons. In Table 1, the *p* value was corrected for the number of analyzed parameters (*n* = 21); in Table 2 and Table 3, it was corrected for the number of analyzed inflammatory markers (*n* = 6). Multiple linear regression models were used to control additional parameters with potential influence on correlation results. The log-transformed values of IM were used in the regression analysis. The models took into account DTI parameters as dependent variables and: (a) the level of particular IM, age, and BMI; or (b) the level of particular IM, age, BMI, smoking, disease duration, exacerbations, and types and doses of antipsychotics as predictors. The sample size was estimated based on assumption, that we would like to be able to detect moderate correlations (ρ > 0.50) between the levels of inflammatory markers and SLF or ILF integrity with a power of 0.80. According to these assumptions, the minimum sample size is 29 [55,56].

The initial number of recruited patients was 39 for CON, 16 for UHR, 30 for FEP, and 74 for SCH. However, after excluding subjects with no information on the levels of IM (no blood drawn or hemolysis, no consent to re-draw blood, or unsuccessful attempts to re-establish contact with the study participant; 8 participants excluded) and/or complete DTI data (a maximum of 2 attempts to perform the examination were made; 41 participants excluded), the final group sizes were 29, 12, 19, and 52, respectively.

The assumption of minimal sample size was met for the HC and SCH groups. Problems with recruitment and failure to complete the DTI examination by some patients resulted in lower numbers of UHR and FEP groups; however, in FEP, they were sufficient to detect highly correlated (ρ > 0.70) measures with the desired power (0.80) (*n* > 12).

## 3. Results

### 3.1. Descriptive Analysis of the Study Groups

The analyzed groups differed in terms of age, BMI, cigarette use, and serum concentrations of IL-6 and IFN-γ. Patient groups additionally differed in duration of illness, number of exacerbations, and the types and doses of antipsychotics. All differences, with the exception of IFN-γ, remained significant after correction for multiple comparisons. Further details on demographic and clinical data for study groups are presented in Table 1.

### 3.2. Analysis of Correlations between Inflammatory Markers and DTI

In HC, the level of IL-8 was negatively correlated with the MD of the right SLF. In UHR, the level of IFN-γ was positively correlated with FA of the left ILF. In FEP, CRP was positively correlated with FA of the left SLF and left ILF. All of these correlations reached *p*-values between 0.01 and 0.05 without correction and none remained significant after correction, for multiple comparisons (Table 2 and Table 3).

Our previous study showed that the levels of some IM parameters ([15], supplementary table) and some DTI parameters of SLF and ILF (data not shown) are correlated with age and BMI. We also found some associations between IM and smoking, disease duration, exacerbations, types, and doses (chlorpromazine equivalent) of antipsychotics ([15], supplementary table). Therefore, for each analyzed IM and DTI parameter, an additional multiple linear regression analysis was performed in two variants: (a) taking into account age and BMI (in every study group); and (b) taking into account age, BMI, smoking, disease duration, exacerbations, types, and doses of antipsychotics (in UHR (without smoking), FEP, and SCH).

Among all analyzed multiple linear regressions, only one model considering IFN-γ, age, and BMI as predictors of MD of left ILF in UHR group was significant (adjusted R^2^ = 0.486, F (3, 8) = 4.47, *p* = 0.04); however, only levels of IFN-γ significantly predicted MD of left ILF (β = −0.725, *p* = 0.01).

We have also found that in the models with age, BMI, and CRP as predictors, the serum level of CRP significantly predicted FA of right SLF in CON (β = 0.499, *p* = 0.041) and FA of left ILF in FEP (β = 0.550, *p* = 0.029); however, these models and other included predictors were not significant (*p* > 0.05). In the models with age, BMI, and IFN-γ as predictors, the levels of IFN-γ significantly predicted FA of left ILF in UHR (β = 0.567, *p* = 0.049) and the models, and other included predictors were not significant (*p* > 0.05). We did not find any significant associations in the models with additional clinical variables as cofactors.

## 4. Discussion

While the associations between schizophrenia and increased levels of inflammatory markers (IM) and between schizophrenia and abnormalities in white matter (WM) microstructure have already been quite well confirmed, knowledge about possible links between these two potential risk factors is limited. Some studies indicate the presence of such associations but differ in the cytokines and WM tracts involved. Our study did not show the existence of such links between the analyzed peripheral IM and the integrity of the superior longitudinal fasciculus (SLF) or inferior longitudinal fasciculus (ILF) in either healthy subjects or patients with schizophrenia spectrum disorders. Despite our broad analysis of six inflammatory markers, taking into account two different markers of WM integrity and both healthy controls and patients at different stages of the schizophrenia spectrum, only four correlations were observed, and they did not remain significant after correction for multiple comparisons. These correlations were not strong, and their *p*-values, without correction, were not lower than 0.01. Moreover, the direction of these correlations was opposite from what was expected. We assumed that increased levels of C-reactive protein (CRP) and proinflammatory cytokines, such as IL-8 and IFN-γ, would be associated with decreased integrity of white matter, presenting as either reduced fractional anisotropy (FA) or increased mean diffusivity (MD). This means that correlations should be negative for FA comparisons and positive for MD (with the exception of anti-inflammatory IL-10); however, we obtained the opposite results. Additionally, the observed correlation was not confirmed in the results for other study groups, inflammatory markers, or markers of WM integrity (FA/MD). In first episode of psychosis patients (FEP), the correlation between CRP and FA was observed for both the left ILF and left SLF, but this observation is likely a result of the correlation between FA of the left SLF and left ILF in FEP (ρ = 0.665, *p* = 0.0019). Similar results were observed in regression analyses. We have used multiple linear regression to control potential confounding factors, such as age, BMI, and some clinical parameters that were found to be associated with the levels of some IM in our previous study. However, despite extensive analysis (in each study group) of models for eight DTI parameters and each IM combined with (a) age and BMI and/or (b) age, BMI, and additional factors as predictors (336 models in total), only a few results showed a particular IM as a significant predictor (without any correction for multiple comparisons). The direction of associations was opposite to what was expected. All of the above arguments indicate the random nature of the observed correlations.

In contrast, Rodrigue et al. [42] found negative correlations between FA of these regions and peripheral levels of Il-6, IL-8, and TNFα in the general population. They took into account age, sex, and BMI as covariates. We did not include sex in our analysis because our previous study did not show any associations between sex and the levels of IM; however, we performed an extensive analysis with age and BMI as covariates and did not find any significant associations between these IM and any of the DTI parameters. However, the study of Rodrigue et al. was carried out on a large sample of extended families, and they found that both cytokine levels and SLF and ILF integrity are significantly heritable, which may have influenced the results.

Prasad et al. [19] analyzed correlations between IL-6 and CRP and the integrity of fronto-thalamic and fronto-temporo-occipital WM tracts in patients with schizophrenia and found that both IL-6 and CRP were negatively correlated with FA of the ILF in schizophrenia patients but not in controls. They do not describe results for the SLF, so we do not know whether there were no correlations in this region or whether it was simply not analyzed. The patient group in the cited study consisted of young adults with schizophrenia or schizoaffective disorder with a mean duration of illness of about 3 years from the first psychotic symptoms, so it does not directly reflect any of our groups. Additionally, the author included sex, age, and socio-economic status as covariates, but they did not take into account current inflammatory diseases as an exclusion factor, which is reflected in the levels of IM that they observed. The mean levels of CRP in both groups were above 15 mg/L, while levels above 10 mg/L are commonly recognized as indicating clinically significant inflammatory processes [57]. The mean levels of IL-6 were about 1 ng/mL, which is about 200 times more than the average levels in healthy individuals [58]. There were no significant differences in these parameters between patients and controls. In comparison to our study, the described parameters indicate a much more intense inflammation both in the control group and in patients in their study. It is difficult to say what the effect of increased inflammation in the participants of the study was on the observed correlation with ILF.

Fu et al. [41] did not find any correlations between inflammatory markers and FA or MD of the SLF or sagittal striatum (including the inferior longitudinal fasciculus and inferior fronto-occipital fasciculus) in schizophrenia patients or the control group, which is in line with our results. The authors focused on IL-10, which was found to be correlated with other WM tracts in their study; however, analyses of other IM, including IL-6, IL-8, and IFN-γ, were also performed and are presented in the supplementary material.

Taking into account the cited papers and our results, it does not seem that current peripheral subclinical inflammation, which is often seen in patients with psychosis, is associated with disruptions of SLF or ILF microstructure in schizophrenia spectrum disorder patients. It is likely that there are other unidentified factors engaged in this process. It is also possible that the reduced integrity of these tracts is related to local inflammation in the central nervous system that is not directly reflected in the levels of cytokines in the peripheral blood or that the reduced integrity is a result of inflammatory processes that occurred in the past. Taking into account the contradictory results of Prasad et al. [19], we should also note the possibility that these associations may be seen in patients with high-level inflammation, at least in the ILF.

The major limitation of our study was the relatively small size of the ultra-high risk and first episode of psychosis groups; therefore, the results in these groups should be considered the pilot study. The sizes of the healthy control and chronic schizophrenia groups were sufficient for the detection of moderate correlations with adequate power, but weak correlations could still go undetected. However, even if such correlations were missed, their clinical significance would probably be small. The second limitation was the small sample size of study groups for multiple regression analyses, taking into account age, BMI, and some clinical variables as potential confounding factors; therefore, we may not have detected some significant associations. We plan future studies with larger sample sizes, in which it will be possible to better control these factors. The third limitation was the lack of opportunity to investigate cause-and-effect dependences due to the cross-sectional nature of the study. Fourth, low spatial resolution at 3T limited the accuracy of the DTI measurements. Fifth, we based the measurements of IM concentrations on single samples. The levels of IM may fluctuate from day to day due to many factors; therefore, the mean levels obtained in serial measurements on different days would probably be better markers of the severity of chronic subclinical inflammation. Finally, we did not take into account some factors that may influence IM levels, such as taking anti-inflammatory medications or having had past infections or autoimmune diseases.

In conclusion, our study does not support the hypothesis that there exist associations between disrupted levels of peripheral inflammatory markers and reduced integrity of the ILF and SLF in schizophrenia spectrum disorders. However, taking into account the limitations of the current study and the contradictory results of available research, we cannot exclude the possibility that disruptions of WM integrity in the SLF and/or ILF are due to subclinical inflammation over a long period of time. Prospective studies combined with repeated measurements of IM levels would be necessary to verify this.

## Figures and Tables

**Table 1:** Demographic, anthropometric, and clinical data for study groups.

Parameter	HC	UHR	FEP	SCH	H/x2
*n*	29	12	19	52	
Women	17 (59)	6 (50)	13 (68)	23 (44)	3.82
Age (years)	36 ± 8 (37)	25 ± 5 (25.5)	27 ± 5 (27)	38 ± 7 (37.5)	41.80 ***#
Years of education	15 ± 3 (15)	13 ± 4 (12)	14 ± 3 (12)	13 ± 3 (12)	5.89
BMI (kg/m^2^)	25 ± 4 (25)	22 ± 3 (22)	24 ± 4 (24)	28 ± 4 (28)	21.24 ***#
Smoking cigarettes	0 (0)	0 (0)	4 (21)	20 (38)	19.33 ***#
Duration of illness (years)		1.07 ± 1.41 (0.5)	0.36 ± 0.37 (0.25)	15.10 ± 5.63 (13.00)	59.40 ***#
Exacerbations ^		4.08 ± 5.85 (1.00)	1.11 ± 0.32 (1)	6.52 ± 4.54 (5)	41.53 ***#
Antipsychotic medications:					
atypical		6 (50)	15 (79)	32 (62)	27.20 ***#
atypical and typical		0 (0)	2 (11)	16 (31)	
typical		0 (0)	1 (5)	2 (4)	
none		6 (50)	1 (5)	2 (4)	
chlorpromazine equivalent (mg)		132 ± 226 (17)	500 ± 345(450)	628 ± 314 (600)	
Antidepressant medications		4 (33)	0 (0)	2 (4)	21.38 ***#
Global functioning in GAF		64 ± 14 (67.5)	61 ± 17 (65)	58 ± 15 (60)	1.94
Psychopathological symptoms in PANSS:					
positive			11 ± 5 (12)	8 ± 4 (6)	2.77 **
negative			14 ±5 (14)	17 ± 6 (15)	−1.38
disorganization			14 ± 4 (13)	12 ± 4 (11)	1.59
affect			10 ± 4 (9)	9 ± 3 (8)	1.93
resistance			6 ± 2 (5)	5 ± 1 (4)	1.96 *
Psychopathological symptoms in SIPS:					
positive		5.8± 3.9 (5)			
negative		10.3 ± 6.1 (11)			
disorganization		3.8± 2.9 (2.5)			
general		7.4 ± 4.0 (7.5)			
CRP (mg/L)	1.55 ± 1.78 (0.86)	3.32 ± 5.62 (0.91)	1.13 ± 1.05 (0.65)	3.01 ± 4.72 (2.05)	7.45
IL-6 (pg/mL)	1.14 ± 0.72 (0.95)	2.12 ± 1.07 (2.19)	1.33 ± 0.53 (1.37)	2.73 ± 3.77 (1.62)	19.46 ***#
IL-8 (pg/mL)	11.29 ± 7.36 (10.15)	10.27 ± 3.66 (9.52)	8.51 ± 4.06 (7.65)	13.21 ± 25.14 (9.49)	5.95
IL-10 (pg/mL)	1.07 ± 0.95 (0.98)	1.03 ± 0.71 (1.14)	7.47 ± 27.46 (0.69)	5.33 ± 25.86 (1.34)	4.45
TNF-α (pg/mL)	6.79 ± 2.54 (6.21)	7.86 ± 4.26 (6.42)	7.06 ± 2.81 (6.09)	6.64 ± 2.52 (6.08)	0.49
IFN-γ (pg/mL)	3.25 ± 4.17 (2.00)	11.67 ± 17.95 (4.63)	4.72 ± 4.99 (3.36)	11.29 ± 35.63 (2.97)	9.88 *

Data are presented as either *n* (%) or mean ± SD (median); * *p* < 0.05, ** *p* < 0.01, *** *p* < 0.001 (without correction for multiple comparisons); # significant differences after correction for multiple comparisons; ^ In the UHR group, exacerbations mean the number of periods with present symptoms; in FEP and SCH, conditions requiring hospitalization (in FEP, there was one patient with 2 exacerbations because of self-reporting of exacerbation with spontaneous partial remission before admission to hospital due to re-exacerbation); HC—healthy controls; UHR—patients with ultra-high risk of developing psychosis; FEP—patients with first episode psychosis; SCH—patients with chronic schizophrenia; BMI—body mass index; GAF—Global Assessment of Functioning; PANSS—Positive and Negative Syndrome Scale; SIPS—Structured Interview for Prodromal Syndromes, CRP—C-reactive protein; IL-6—interleukin-6; IL-8—interleukin-8; IL-10—interleukin-10; TNF-α—tumor necrosis factor-α; IFN-γ—interferon-γ.

**Table 2:** Spearman rank correlation coefficients (ρ) for inflammatory markers, fractional anisotropy (FA), and mean diffusivity (MD) of the left and right superior longitudinal fasciculus (SLF) in all analyzed groups.

	HC (*n* = 29)		UHR (*n* = 12)		FEP (*n* = 19)		SCH (*n* = 52)	
FA of SLF	Left	Right	Left	Right	Left	Right	Left	Right
CRP	−0.060	0.176	−0.063	−0.210	0.489 *	0.344	0.141	0.203
IL-6	−0.209	0.019	−0.315	−0.224	0.298	0.074	−0.004	0.008
IL-8	0.086	0.179	−0.217	−0.371	0.014	0.174	0.209	0.112
IL-10	0.256	0.305	0.053	0.049	−0.159	0.063	0.003	−0.224
TNF-α	−0.039	0.264	0.259	0.420	−0.177	−0.239	0.076	0.163
IFN-γ	−0.297	−0.175	−0.084	−0.224	−0.044	0.057	0.120	−0.074
MD of SLF	Left	Right	Left	Right	Left	Right	Left	Right
CRP	−0.113	−0.310	−0.266	0.063	0.177	−0.032	0.255	0.208
IL-6	0.054	−0.023	−0.063	0.210	0.123	0.100	0.041	−0.009
IL-8	−0.180	−0.400 *	−0.168	0.308	−0.309	−0.307	−0.208	−0.153
IL-10	0.096	0.157	0.053	−0.284	0.178	−0.152	0.132	0.151
TNF-α	−0.019	−0.116	−0.161	−0.252	0.167	−0.037	0.076	0.049
IFN-γ	0.033	0.112	−0.014	0.140	−0.046	0.127	−0.005	0.169

* *p* < 0.05 (without correction for multiple comparisons, no statistically significant correlations after correction).

**Table 3:** Spearman rank correlation coefficients (ρ) for inflammatory markers, fractional anisotropy (FA), and mean diffusivity (MD) of the left and right inferior longitudinal fasciculus (ILF) in all analyzed groups.

	HC (*n* = 29)		UHR (*n* = 12)		FEP (*n* = 19)		SCH (*n* = 52)	
FA of ILF	Left	Right	Left	Right	Left	Right	Left	Right
CRP	0.041	0.081	0.133	0.238	0.518 *	0.207	−0.103	−0.107
IL-6	−0.191	−0.138	−0.175	0.238	0.182	0.056	0.088	0.110
IL-8	−0.006	−0.237	−0.266	−0.294	0.070	0.088	0.142	0.129
IL-10	0.008	−0.122	0.294	0.130	0.071	−0.076	0.016	−0.087
TNF-α	0.220	0.117	−0.287	0.238	−0.040	−0.382	−0.058	0.093
IFN-γ	0.110	0.024	0.608 *	0.280	0.211	0.006	−0.019	0.021
MD of ILF	Left	Right	Left	Right	Left	Right	Left	Right
CRP	−0.088	−0.114	0.028	−0.056	−0.049	−0.002	0.188	0.237
IL-6	0.149	0.135	−0.014	−0.427	0.089	0.163	−0.072	0.026
IL-8	−0.097	0.061	−0.168	0.161	−0.374	−0.291	−0.053	−0.067
IL-10	0.214	0.167	−0.375	−0.462	−0.160	−0.256	−0.027	0.140
TNF-α	−0.101	−0.107	−0.070	−0.238	−0.354	−0.177	0.022	−0.003
IFN-γ	−0.195	−0.005	−0.531	−0.392	−0.397	−0.168	−0.017	0.012

* *p* < 0.05 (without correction for multiple comparisons, no statistically significant correlations after correction).

## Data Availability

Data are available from the corresponding author on reasonable request.
